# Local recurrences at the anastomotic area are clonally related to the primary tumor in sporadic colorectal carcinoma

**DOI:** 10.18632/oncotarget.17200

**Published:** 2017-04-18

**Authors:** Efsevia Vakiani, Ronak H. Shah, Michael F. Berger, Alvin P. Makohon-Moore, Johannes G. Reiter, Irina Ostrovnaya, Marc A. Attiyeh, Andrea Cercek, Jinru Shia, Christine A. Iacobuzio-Donahue, David B. Solit, Martin R. Weiser

**Affiliations:** ^1^ Department of Pathology, Memorial Sloan-Kettering Cancer Center, New York, NY, 10065, USA; ^2^ Human Oncology and Pathogenesis Program, Memorial Sloan Kettering Cancer Center, New York, NY, 10065, USA; ^3^ The David Rubenstein Pancreatic Cancer Research Center, Memorial Sloan Kettering Cancer Center, New York, NY, 10065, USA; ^4^ Program for Evolutionary Dynamics, Harvard University, Cambridge, MA, 02138, USA; ^5^ Department of Epidemiology and Biostatistics, Memorial Sloan-Kettering Cancer Center, New York, NY, 10065, USA; ^6^ Department of Medicine, Memorial Sloan-Kettering Cancer Center, New York, NY, 10065, USA; ^7^ Department of Surgery, Memorial Sloan-Kettering Cancer Center, New York, NY, 10065, USA

**Keywords:** next-generation sequencing, colorectal cancer, anastomotic recurrence

## Abstract

**Purpose:**

Anastomotic recurrences (AR) occur in 2–10% of colorectal carcinoma cases after resection of primary tumor (PT). Currently, there are no molecular data investigating their genetic profile and multiple theories exist about their pathogenesis. The aim of our study was to compare the genomic profile of AR to that of the patients’ corresponding matched PT and, when available, to a distant metastasis (DM).

**Experimental Design:**

Thirty-six tumors from 14 patients were genotyped using a capture-based, next-generation assay to define the mutational status of 341 cancer-associated genes. All patients had R0 resection of their PT and AR occurred 1.1−7.0 years following PT resection. A DM or a second AR was analyzed in 8 patients. All tumors were microsatellite stable except in one patient with Lynch syndrome.

**Results:**

A total of 254 somatic mutations were detected including 138 mutations in the microsatellite stable (MSS) cases. The most commonly mutated genes were *APC*, *KRAS*, *TP53*, *PIK3CA*, *ATM* and *PIK3R1*. In all patients with MSS tumors the AR and PT shared between 50−100% of mutations, including mutations in key driver genes, consistent with these tumors being clonally related. Genetic events private to DM were not detected in AR and phylogenetic analysis showed that ARs were more closely related to PT than DM. In the Lynch syndrome patient the PT and AR showed distinct somatic mutations consistent with independent primaries.

**Conclusions:**

ARs are clonally related to PT in sporadic colorectal carcinomas and do not appear to represent seeding of the anastomotic site by distant metastases.

## INTRODUCTION

Colorectal cancer (CRC) is the fourth most common malignancy in the United States and second most common cause of death with an estimated 49,000 deaths in 2016 [1]. When diagnosed at an early stage it can be surgically cured, although 40% of patients will have a local or distant recurrence after “curative” surgery [2]. Local recurrence (LR) is defined as tumor in and around the tumor bed including peri-colic fat, adjoining mesentery and lymph nodes (extramural recurrence) or in the suture or staple line of the anastomosis (intramural/anastomotic recurrence) [3]. Most cases of LR occur within 2 years of surgery with a peak between 6–12 months and where possible are treated with salvage resection as this has been associated with an improved outcome [3, 4].

Several theories exist regarding the pathogenesis of LRs. They include regrowth of residual malignant cells left behind locally after curative surgery, seeding by disseminated cells, and a second primary tumor (PT) in an area of proliferative instability [5–7]. Indeed it is possible that several mechanisms are at play and that they relate to the site of recurrence. Some hypotheses are put forth mainly in the context of anastomotic recurrences (AR) [6]. For example, it is suggested that many ARs result from exfoliated malignant cells that are implanted into the anastomotic line, a process that is facilitated by altered biological properties at a site of tissue injury. Many surgeons accept this theory and employ intraluminal irrigation where possible to decrease the risk of LR [8]. ARs are also more likely to be clinically considered second primaries, especially when they occur several years following resection of the PT [5].

Most studies exploring the pathogenesis of ARs were performed several decades ago and to date there is no data on the molecular profile of ARs. In the current study, we examine the genetic profiles of recurrent CRC at the anastomotic area in comparison to PT and, where available, a distant metastasis (DM) in order to gain insight into the mechanisms of their pathogenesis.

## RESULTS

### Patient demographics and pathological data

#### Characteristics of patients and primary tumors

Clinicopathologic data are summarized in Table 1 and individual patient and tumor characteristics are shown in Supplementary Table 1. The majority of PTs were located at or distal to the splenic flexure and 8/14 (57%) tumors were located in the rectum (*n* = 3) or sigmoid colon (*n* = 5). PTs were resected with clear margins in all cases, although in 1 patient the tumor was 0.5 mm from the radial margin. Four of the 14 patients presented with oligometastatic/stage IV disease which was completely resected. Eleven of the 14 (79%) patients received adjuvant chemotherapy. Two patients died of their disease, 4 were alive with disease and 8 did not show evidence of disease at last follow-up (mean follow-up time 3.3 years from first AR).

**Table 1:** Clinicopathologic features of study patients (N = 14)

Variable	*N* (%)
Age at diagnosis, yrs	
Mean, Median, Range	60, 61, 46–76
Time between primary tumor and AR, yrs	
Mean, Median, Range	3.1, 2.6, 1.1–7
Sex	
F	6
M	8
Site of primary tumor	
Right Colon	4
Splenic flexure	2
Sigmoid	5
Upper rectum	3
Stage at diagnosis of primary tumor	
I	1
II	7
III	2
IV	4
Distance metastasis at time of AR	
Yes	3
No	11
Interval treatment between PT and AR	
Yes	12
No	2
Outcome	
DOD	2
AWD	4
NED	8

PT: Primary tumor; AR: Anastomotic recurrence.

In 12/14 (86%) cases the PT was moderately differentiated and in 2 poorly differentiated. Two tumors had > 50% extracellular mucin and were classified as mucinous, while 2 had mucinous features (< 50% extracellular mucin). One poorly differentiated adenocarcinoma with mucinous features showed morphologic heterogeneity in addition to increased tumor intraepithelial lymphocytes and was found to have lost expression of MLH1 and PMS2. This patient was later confirmed to have a deleterious germline mutation in the *MLH1* gene (Lynch syndrome). Among the remaining 13 PTs no abnormalities were detected in the expression of mismatch repair proteins.

#### Characteristics of anastomotic recurrences

ARs occurred 1.1 to 7 years (mean 3.1, median 2.6 years) following resection of the PT. In 11 (79%) patients the AR was detected on surveillance colonoscopy while in the remaining 3 patients it was detected on imaging and in one of these 3 patients confirmed with colonoscopy. In 7/12 (58%) patients an exophytic tumor was seen during colonoscopy, while in the remaining 5 patients the endoscopist noted inflammation and granularity (*n* = 2), stenosis (*n* = 1), ulcer (*n* = 1) or firmness (*n* = 1). In 9 patients the recurrent tumor was right at the anastomotic line, in 2 patients with a sigmoid PT it was 5 and 8 cm proximal to the anastomotic line, while in 3 patients with a sigmoid (*n* = 2) or upper rectal PT it was 4, 6, and 8 cm distal to the anastomotic line. Eleven patients underwent R0 resection of the AR (after radiation in one case/pt 11), while in 3 patients the AR was only biopsied and not resected for a variety of reasons including tumor location, patient age or presence of unresectable distant metastatic disease.

In 13 (93%) patients the histology of the AR was the same as that of the PT. In the patient with Lynch syndrome the AR was histologically different compared to the PT and showed a moderately differentiated adenocarcinoma without a mucinous component. In 9 (64%) patients (including the Lynch syndrome patient) the AR showed an adenoma-like component raising the possibility of a new PT on histologic grounds alone.

#### Characteristics of second anastomotic recurrences and distant metastases

Additional samples were genotyped in 8 patients. A DM that occurred 1.2–3 yrs prior to the AR was profiled in 5 patients (liver *n* = 3, lung *n* = 1, abdomen *n* = 1). The metastasis occurred at the same time as the PT in 1 patients while in 4 patients it occurred 4–48 months (pt 5) and 4 years (pt 6) after resection of the primary tumor. In 3 patients a second AR was also included in the study. These second ARs occurred 19, 22 and 27 months following resection of the first AR, were detected on surveillance colonoscopy and were subsequently completely resected. They occurred at the anastomotic line in 2 cases and 2 cm distal to the anastomotic site in 1 case (pt 9). Their endoscopic and histologic characteristics were similar to those seen in the first AR.

### Massive parallel sequencing

#### Patterns in mutational spectrum

**Figure 1:**
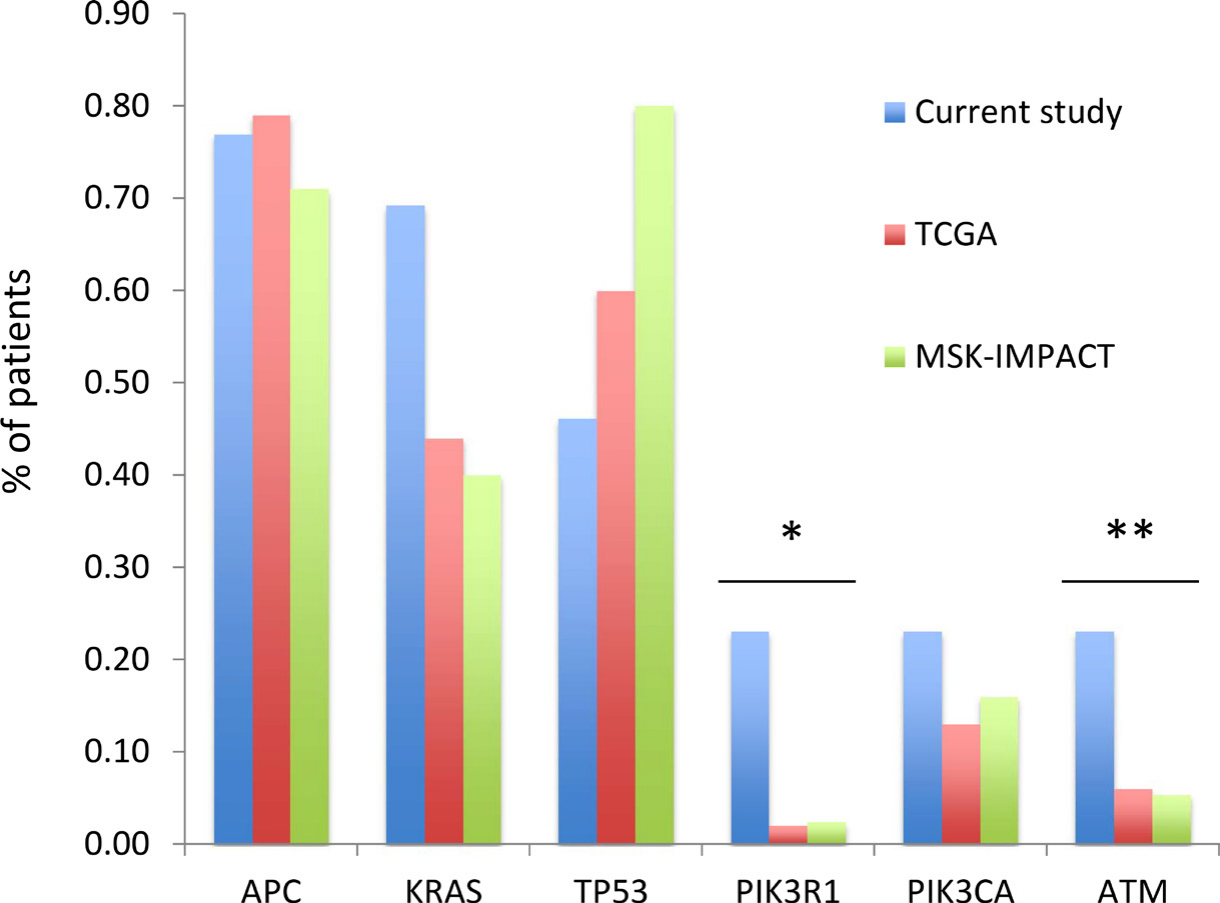
Frequency of mutations in the current series compared to that seen in the TCGA and clinical MSK-IMPACT cohorts Only microsatellite stable cases are shown.*p < 0.01, **p = 0.04.

A total of 254 nonsynonymous mutations were detected, 116 of which were present in the 2 microsatellite unstable (MSI-H) tumors from the patient with Lynch syndrome (pt 7). Among the 34 microsatellite stable (MSS) samples a total of 138 distinct non-synonymous mutations were detected including 92 missense mutations and 46 truncating mutations (nonsense *n* = 23, splice site *n* = 4, insertions/deletions *n* = 19). Among founder mutations 6 genes were noted to be altered in 3 or more patients with MSS tumors (Figure 1). Mutations in *APC* and *PIK3CA* were present in a similar number of patients compared to what was observed in the TCGA and clinical MSK-IMPACT cohorts. *KRAS* mutations were seen in a higher number of patients, though the difference was not statistically significant (69% vs 44%, *p* = 0.1). In contrast, *TP53* was altered in a significantly smaller number of patients (46% vs 60% and 80% in the TCGA and MSK-IMPACT clinical series, *p* < 0.01). Interestingly, the *ATM* and *PIK3R1* genes that were mutated in less than 10% of MSS cases in the TCGA and clinical MSK-IMPACT cohorts were among the 6 most frequently mutated genes in this study. Both genes were altered in 3 (23%) patients compared to 6% of patients in the TCGA and MSK-IMPACT cohorts for *ATM* (*p* = 0.04) and 2% of patients for *PIK3R1* (*p* < 0.01). Notably, all mutations in these two genes were either truncating or recurrent hotspots.

#### Clonal relationship of anastomotic recurrences to primary tumor

**Figure 2:**
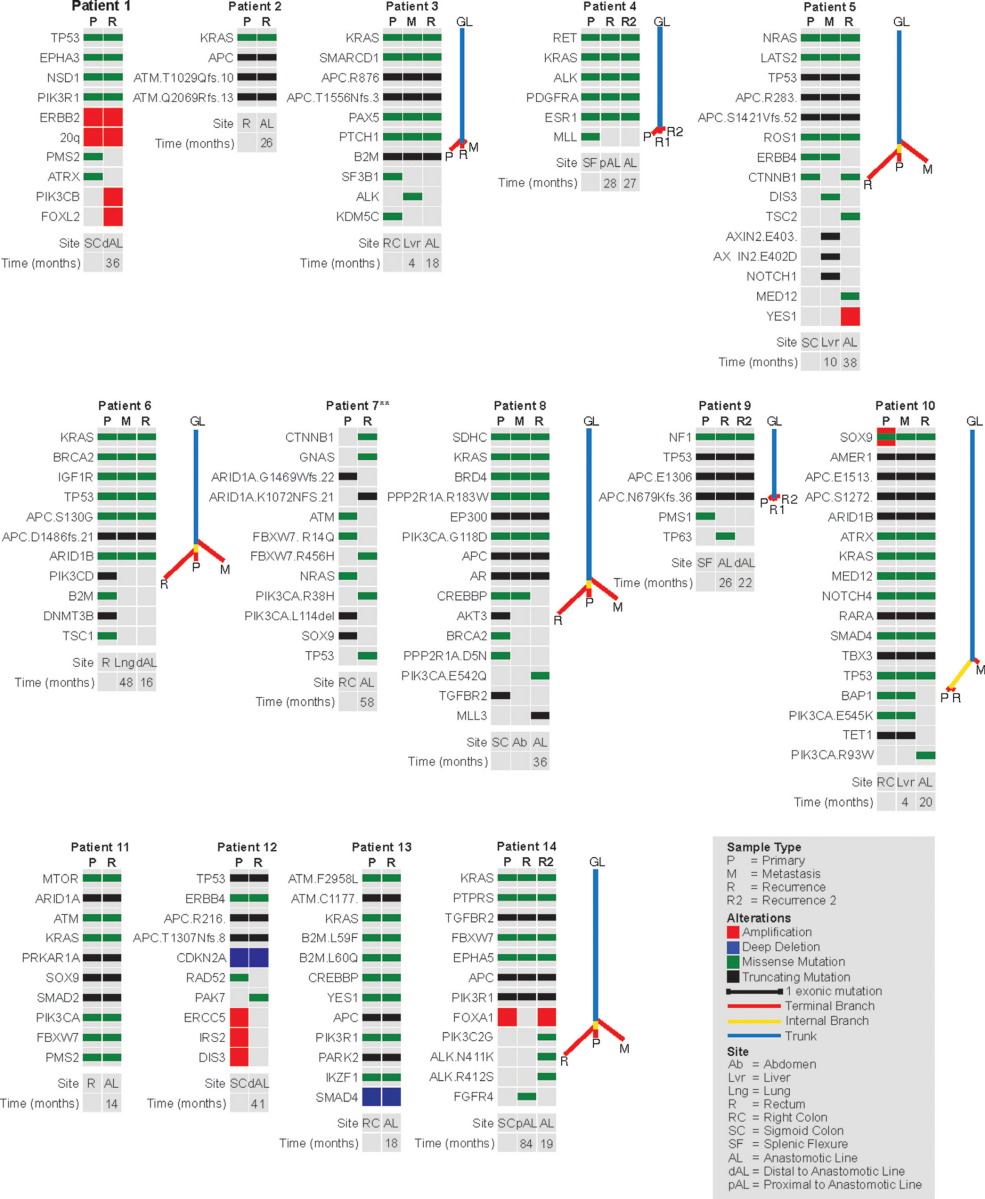
Heatmaps showing the distribution of mutations and copy number alterations in primary tumors, anastomotic recurrences and distant metastases Phylogenetic trees are shown to the right of the heatmaps for cases where more than 2 samples were genotyped. ** for patient 7 only a subset of mutations are shown due to space constraints.

Primary MSS tumors and first ARs showed 127 distinct mutations and among them 106 (83%) were shared between the 2 tumors. In each of the 13 patients there were at least 4 genetic events present in both the PT and the AR, consistent with the 2 tumors being clonally related (Figure 2). Mutations in recurrently altered genes in MSS CRC (altered in > 10% of cases in the TCGA study) were always concordant between the PT and the AR. AR private mutations appeared to be subclonal with a mean variant allele frequency (VAF) of 0.13 (vs 0.32 VAF for shared mutations), were not among recurrent cancer mutations [9] and did not have known functional consequences, although truncating mutations might be expected to have deleterious effects. Overall, these observations support the hypothesis that ARs originate from the dominant clone in the PT and show subsequent subclonal evolution.

In the Lynch syndrome patient (pt 7) both the PT and the AR had over 50 mutations each as expected for MSI-H tumors. Contrary to what we observed in the MSS cases none of the genetic events were shared between the 2 tumors with the exception of a one base pair deletion in *RNF43* present with a 16% VAF in AR and a 2% VAF in the PT. This alteration was in a homopolymeric tract and as such could occur in unrelated MSI-H tumors by chance. Therefore, our findings in this case are consistent with the AR representing a new primary CRC.

A second AR that occurred 1.7–2.2 yrs after the first AR was analyzed in 3 patients. In these patients the clinical impression as documented in the medical record was of multiple distinct primaries raising the possibility of an underlying genetic abnormality that might predispose to multiple CRCs. In all 3 patients, we found that the second AR was clonally related to the respective prior tumors and did not represent a different primary. In one patient the 3 tumors showed no differences, while in the other two patients (pts 9 & 14) the second AR showed a single novel genetic event compared to PT.

#### Clonal relationship of distant metastases to anastomotic recurrences

A DM that occurred 1.2–3 years prior to the AR was profiled in 5 patients. These metastases were all clonally related both to the PT and the AR as evidenced by the presence of shared genetic alterations in multiple genes. In 4/5 patients we found a total of 10 genetic events (6 missense mutations, 3 truncating mutations, 1 amplification) that were private to DM. In agreement with prior observations [10, 11], *PIK3CA* was the only gene among the recurrently altered genes in CRC to show genetic heterogeneity between PT and DM. Most of the private mutations identified in the metastases (7/9) were subclonal (mean VAF 0.11 for private mutations vs 0.35 for shared mutations), although the PIK3CA E545Q and MLL3 mutations (pt 8) appeared clonal. None of the private events in the metastases were detected in the ARs and phylogenetic analysis in these cases indicated that the ARs were more closely related to PT than to DM (Figure 2). Overall, these observations argue against the hypothesis that tumor cells from DM seed anastomotic sites giving rise to AR.

## DISCUSSION

Recurrences at the anastomotic area occur in a small number of CRC patients and are associated with significant morbidity and mortality [4, 12]. Their reported incidence varies from 2.7% to 11.7% in part due to differing surgical techniques as well as difficulties in precise identification of the recurrence site [4, 13–18]. The literature on ARs is limited and they are often discussed in the context of locally recurrent disease, although their clinical and biological properties might differ from extramural recurrences.

Metachronous carcinogenesis has been proposed as the most plausible explanation for late ARs with “late” having been arbitrarily defined by some authors as those ARs that occur at least 2 years following resection of the primary tumor [5, 7]. The possibility of independent primaries is supported by the field defect theory according to which the bowel mucosa in some patients is more prone to developing multiple neoplastic lesions [19–21]. Moreover, the increased yield of bowel tumors at the site of transection, found consistently in animal studies [22–24], has led to the hypothesis that anastomotic sites are also more susceptible to *de novo* neoplasia in humans, especially in the context of a field defect. Our study is the first to explore the possibility of independent primaries on a molecular level. Independent PTs were detected only in the single Lynch patient included in our study, which is consistent with the observation that patients with Lynch syndrome carry a significant risk of metachronous CRC [25]. In contrast, we did not find evidence for metachronous primaries in any of the sporadic, MSS tumors including cases where the AR occurred more than 2 years following resection of the PT.

The finding of clonally related tumors in 13 of 14 patients is consistent with the presence of residual malignant cells derived from the dominant clone in the PT that have the ability to grow at the anastomotic area. The reservoir of these malignant cells and the mechanism by which they grow and give rise to an AR remains unknown. Implantation of exfoliated cells at the site of tissue injury is considered to be one of the etiological mechanisms of ARs. The observation that ARs often show an adenoma-like component, as was the case in 9 tumors in the present study, might argue in favor of a mucosa to bowel wall direction of events that would be consistent with the theory of exfoliated cell implantation [7, 17]. Nonetheless, colonization of basement membranes by underlining invasive carcinomas mimicking a precursor lesion occurs in many tissue types and, therefore, one cannot exclude an extraluminal source of malignant cells even in cases showing adenoma-like lesions. Our molecular data would be equally compatible with the presence of residual malignant cells within lymphovascular spaces.

We did not find evidence to support the hypothesis that tumors cells that have established a DM re-enter the circulation and seed the anastomotic area. In 5 patients where we sequenced a DM that was resected prior to the AR we did not detect any metastasis-private genetic events within the AR. This does not argue against the seeding of anastomotic sites by circulating tumor cells prior to them undergoing clonal evolution within distant sites of metastases. Moreover, comparison of ARs to DMs in the current study is based on a relatively small number of genetic events and it is possible that a more complicated relationship might emerge using multi-region and/or whole exome sequencing.

We found a higher frequency of mutations in *PIK3R1* and *ATM* compared to what was observed in the TCGA and clinical MSK-IMPACT cohorts and all the observed mutations were either truncating or recurrent hotspots [9] and as such would be expected to have deleterious consequences. The *PIK3R1* gene mutations were in the iSH2 domain and these mutations have been shown *in vitro* to promote cell survival, AKT activation and anchorage independent cell growth [26]. Less is known about the effects of *ATM* mutations found in various tumor types and the mechanisms by which they might promote oncogenesis. The *ATM* gene is best known as an activator of the DNA damage response following DNA double stranded breaks, but it appears to be involved in a number of other signaling pathways that affect cell growth [27, 28]. Our findings raise the possibility that certain genetic events might be more likely to promote the survival of malignant cells in their local environment with subsequent re-growth at the anastomotic area. This hypothesis would need to be explored in future larger studies, as our study group is small and highly selected with many patients having relatively indolent disease. It is unlikely, however, that *PIK31R1* and *ATM* mutations are markers of indolent disease in general as the frequency rates did not differ between the TCGA cohort and the MSK-IMPACT clinical cohort, the latter being composed predominantly of stage IV patients with inferior outcomes.

In conclusion, our study is the first to examine the molecular profile of ARs and to show that in sporadic CRC ARs are clonally related to PTs. We did not find evidence to suggest that ARs are the result of disseminated disease and our data would be most consistent with the presence of a local reservoir of malignant cells able to re-grow at the anastomotic area. This hypothesis is also in line with the clinical experience showing that complete surgical resection of ARs is associated with an excellent prognosis. Future larger studies are needed to confirm our findings.

## MATERIALS AND METHODS

### Patient and tumor characteristics

This study was approved by the Institutional Review Board at our institution. We identified 14 patients who developed a recurrence at or close to the anastomosis at least one year following R0 resection of a primary CRC with adequate tissue available both from the PT and the recurrence for genomic analysis. Five patients had tissue available from a DM that developed prior to the AR. Three patients had tissue available from a second AR that occurred after the first AR. A total of 36 samples were analyzed.

Pathology data were recorded after reviewing all hematoxylin & eosin (H&E) slides and clinical data were collected through review of the electronic medical records under an IRB-approved waiver of authorization. Tumors that were located 15 cm or less from the anal verge were classified as rectal. Microsatellite instability was assessed by immunohistochemistry (IHC) using antibodies against the mismatch repair proteins MLH1 (clone G168-728, diluted 1:250, BD PharMingen, San Diego, CA), MSH2 (clone FE11, diluted 1:50, Oncogene Research Products, Cambridge, MA), MSH6 (clone GRBP.P1/2.D4, diluted 1:200; Serotec Inc., Raleigh, NC), and PMS2 (clone A16-4, diluted 1:200, BD PharMingen, San Diego, CA). Tumors showing loss of expression in one or more of these proteins were designated as microsatellite unstable (MSI-H).

### Next generation sequencing

H&E-stained slides from tumor blocks were reviewed and, if needed, samples were dissected to enrich for tumor content. Normal colorectal mucosa away from tumor was used as a source of germline DNA. DNA from normal and tumor blocks was extracted using the Qiagen DNeasy Blood & Tissue Kit (Qiagen, Valencia, CA), according to standard methods. Massive parallel sequencing was performed using MSK-IMPACT (Memorial Sloan Kettering Integrated Mutation Profiling of Actionable Cancer Targets), a custom next-generation sequencing assay that interrogates the mutational status of 341 cancer-associated genes [29]. Sequence read alignment, processing, single-nucleotide variant and copy number detection were performed as previously described [29]. All variant calls and allele counts were manually reviewed. In cases where a somatic variant was not initially called in all samples from the same patient the event was considered present if the allele count was found to be at least 2% with at least 3 reads after manual review (this occurred in 3/138 variants in the MSS cases). Genomic alterations were considered as founder if present in all samples from the same patient, private if only present in one sample and shared if present in 2 of 3 samples.

Mutation frequencies in the microsatellite stable (MSS) tumors were compared to those seen in the MSS CRC cases in the TCGA cohort (*n* = 189) as well as to those seen in CRC patients who underwent single-site tumor sequencing for clinical purposes at our institution (*n* = 166) [30, 31].

### Phylogenetic and statistical analysis

For each patient with 3 samples, a phylogenetic tree was inferred using Treeomics, an evolutionary analysis for reconstructing tumor phylogenies using a Bayesian inference model [32, 33]. Treeomics determines the probability that a particular variant is either present or absent in a given sample using a statistical approach to account for sequencing errors and varying neoplastic cellularity across the tumors [33]. Each phylogenetic tree was “rooted” at the patient-matched normal sample, and the nodes represented tumor samples. Statistical analysis was performed using chi-square and Fisher's exact tests with two-tailed *p* values.

## SUPPLEMENTARY TABLE





## Abbreviations

CRC: colorectal cancer
AR: anastomotic recurrence
PT: primary tumor
DM: distant metastasis
LR: local recurrence
