# Supplementary material for: Local recurrences at the anastomotic area are clonally related to the primary tumor in sporadic colorectal carcinoma
Source: Oncotarget. 2017 Apr 18;8(26):42487–94. doi: 10.18632/oncotarget.17200 (PMC5522082; doi:10.18632/oncotarget.17200)
Supplement: Supplementary file 1 [file oncotarget-08-42487-s001.pdf]

Local recurrences at the anastomotic area are clonally related to the primary tumor in sporadic colorectal carcinoma

Supplementary Materials

Supplementary Table 1: Detailed clinicopathologic features of patients and samples analyzed. See Supplementary_Table_1
