# Supplementary material for: Local recurrences at the anastomotic area are clonally related to the primary tumor in sporadic colorectal carcinoma
Source: Oncotarget. 2017 Apr 18;8(26):42487–94. doi: 10.18632/oncotarget.17200 (PMC5522082; doi:10.18632/oncotarget.17200)
Supplement: Supplementary file 2 [file oncotarget-08-42487-s002.docx]

| **Supplemental Table 1.** Detailed clinicopathologic features of patients and samples analyzed | | | | | | | | | | | | | | | | | | | | | | |
| --- | --- | --- | --- | --- | --- | --- | --- | --- | --- | --- | --- | --- | --- | --- | --- | --- | --- | --- | --- | --- | --- | --- |
|  | Anastomotic recurrences | | | | | | | | |  |  |  |  |  |  |  |  |  |  |  |  |  |
| Patient | Type of Specimen | Recurrence detected by | Distance from anastomotic line (cm) | Presence of exophytic component during colonoscopy | Presence of Adenoma | Histology of recurrent tumor | Presence of radiation prior to recurrence | Presence of treatment prior to first recurrence | Distant disease at time of recurrence | Gender | Age (yrs) | MSI-H | Site of Primary Tumor | Histology of primary tumor | Distance of primary tumor to closest margin (cm) | Stage of primary tumor | Time between primary and recurrence | Site of distant metastasis sequenced | Time between recurrences (months) | Time between distant metastasis and recurrence | Status at last follow-up | Follow-up time from recurrence (years) |
| 1 | Biopsy | SC | 8, distal | no | no | MD ACA, NOS | no | yes | no | F | 76 | no | sigmoid | MD ACA, NOS | 2.1 | II | 3.0 | NA | NA | NA | AWD | 4.5 |
| 2 | Resection | SC | 0 | no | no | MD ACA, NOS | no | yes | yes | M | 63 | no | rectum | MD ACA, NOS | 2.1 | II | 2.1 | NA | NA | NA | DOD | 2.3 |
| 3 | Resection | Imaging | 0 | NA | yes | MD ACA, NOS | no | yes | no | F | 46 | no | right | MD ACA, NOS | 4.5 | IV | 1.8 | liver | NA | 1.5 | NED | 3.7 |
| 4 | Resection | SC | 5, proximal | yes | no | MD ACA, NOS | no | no | no | F | 68 | no | splenic flexure | MD ACA, NOS | 10 | II | 2.2 | NA | 27 | NA | NED | 4.8 |
| 5 | Resection | SC | 0 | yes | yes | MD ACA, NOS | no | yes | no | M | 60 | no | sigmoid | MD ACA, NOS | 0.05 | II | 4.0 | liver | NA | 3.1 | DOD | 2.5 |
| 6 | Biopsy | Imaging | 4, distal | no | yes | MD ACA, NOS | no | yes | yes | M | 52 | no | rectum | MD ACA, NOS | 1.5 | II | 5.2 | lung | NA | 1.2 | AWD | 0.6 |
| 7 | Biopsy | SC | 0 | yes | yes | MD ACA, NOS | no | no | no | M | 47 | yes | right | PD ACA with mucinous features | 7 | II | 4.8 | NA | NA | NA | NED | 1.8 |
| 8 | Resection | Imaging | 0 | NA | no | MD ACA, NOS | no | yes | no | M | 56 | no | sigmoid | MD ACA, NOS | 1.5 | IV | 3.0 | abdomen | NA | 3.0 | NED | 2.2 |
| 9 | Resection | SC | 0 | no | no | MD to PD ACA, NOS | no | yes | no | M | 49 | no | splenic flexure | PD ACA, NOS | 8.5 | III | 2.1 | NA | 22 | NA | NED | 5.8 |
| 10 | Resection | SC | 0 | yes | yes | MD ACA, NOS | no | yes | no | F | 72 | no | right | MD ACA with mucinous features | 5 | IV | 1.5 | liver | NA | 1.2 | NED | 3.4 |
| 11 | Resection | SC | 0 | yes | yes | MD ACA, NOS | yes | yes | no | F | 62 | no | rectum | MD ACA, NOS | 0.5 | I | 1.1 | NA |  | NA | NED | 6.4 |
| 12 | Excisional Biopsy | SC | 6, distal | yes | yes | MD ACA, NOS | no | yes | no | M | 56 | no | sigmoid | MD ACA, NOS | 1.8 | III | 3.4 | NA | NA | NA | AWD | 1.9 |
| 13 | Resection | SC | 0 | no | yes | Mucinous MD ACA | no | yes | yes | M | 66 | no | cecum | Mucinous MD ACA | 5.5 | II | 1.5 | NA | NA | NA | AWD | 2.9 |
| 14 | Resection | Imaging | 8, proximal | yes | yes | Mucinous MD ACA | no | yes | no | F | 63 | no | sigmoid | Mucinous MD ACA | 9.5 | IV | 7.0 | NA | 19 | NA | NED | 3.6 |
| SC, surveillance colonoscopy; MD, moderately differentiated; PD, poorly differentiated; ACA, adenocarcinoma; NOS, not otherwise specified; NA, non-applicable; NED, no evidence of disease; AWD, alive with disease; DOD, dead of disease | | | | | | | | | | | | | | | | | | | | | | |
